# Plant Growth-Promoting Activity of *Pseudomonas aeruginosa* FG106 and Its Ability to Act as a Biocontrol Agent against Potato, Tomato and Taro Pathogens

**DOI:** 10.3390/biology11010140

**Published:** 2022-01-14

**Authors:** Farideh Ghadamgahi, Saeed Tarighi, Parissa Taheri, Ganapathi Varma Saripella, Alice Anzalone, Pruthvi Balachandra Kalyandurg, Vittoria Catara, Rodomiro Ortiz, Ramesh Raju Vetukuri

**Affiliations:** 1 Department of Plant Protection, Faculty of Agriculture, Ferdowsi University of Mashhad, Mashhad 9177948974, Iran; farideh.ghadamgahi@slu.se (F.G.); p-taheri@um.ac.ir (P.T.); 2 Department of Plant Breeding, Swedish University of Agricultural Sciences, Alnarp, SE-234 22 Lomma, Sweden; ganapathi.varma.saripella@slu.se (G.V.S.); pruthvi.balachandra@slu.se (P.B.K.); rodomiro.ortiz@slu.se (R.O.); Ramesh.Vetukuri@slu.se (R.R.V.); 3 Department of Agriculture, Food and Environment, University of Catania, 95124 Catania, Italy; alice.anzalone@phd.unict.it (A.A.); vcatara@unict.it (V.C.)

**Keywords:** bacterial endophytes, biocontrol, bio-stimulants, plant growth, strain FG106

## Abstract

**Simple Summary:**

Microbial bio-stimulants are attracting increasing attention in agricultural research. In particular, plant growth-promoting rhizobacteria (PGPR) have great potential to improve crops’ productivity and tolerance of biotic and abiotic stresses. It is anticipated that PGPR could eventually replace synthetic fungicides in agriculture. This research evaluated *Pseudomonas aeruginosa* strain FG106—which was isolated from tomato plants– as a potential biocontrol agent against several plant pathogens. This strain displayed multiple plant growth-promoting attributes and high in vitro and in vivo inhibition of growth and pathogenicity of tested phytopathogens. It is thus a multifunctional PGPR with potential applications as a biocontrol agent to control fungal and bacterial pathogens.

**Abstract:**

*P. aeruginosa* strain FG106 was isolated from the rhizosphere of tomato plants and identified through morphological analysis, 16S rRNA gene sequencing, and whole-genome sequencing. In vitro and in vivo experiments demonstrated that this strain could control several pathogens on tomato, potato, taro, and strawberry. Volatile and non-volatile metabolites produced by the strain are known to adversely affect the tested pathogens. FG106 showed clear antagonism against *Alternaria alternata*, *Botrytis cinerea*, *Clavibacter michiganensis* subsp. *michiganensis*, *Phytophthora colocasiae*, *P. infestans*, *Rhizoctonia solani*, and *Xanthomonas euvesicatoria* pv. *perforans*. FG106 produced proteases and lipases while also inducing high phosphate solubilization, producing siderophores, ammonia, indole acetic acid (IAA), and hydrogen cyanide (HCN) and forming biofilms that promote plant growth and facilitate biocontrol. Genome mining approaches showed that this strain harbors genes related to biocontrol and growth promotion. These results suggest that this bacterial strain provides good protection against pathogens of several agriculturally important plants via direct and indirect modes of action and could thus be a valuable bio-control agent.

## 1. Introduction

Tomato, *Solanum lycopersicum* L. (Solanaceae), is a major crop that is widely used as a model for fruit development [1]. It is attacked by several pathogens including *Alternaria alternata*, *Botrytis cinerea*, *Clavibacter michiganensis* subsp. *michiganensis*, *Phytophthora infestans*, *Rhizoctonia solani*, *Xanthomonas euvesicatoria* pv. *perforans*, etc. [2]. These pathogens are significant limiting factors in the production of tomato crops in greenhouses and cause yield losses, especially at the fruiting stage [3].

Current methods for managing harmful plant pathogens depend mainly on the extensive use of synthetic chemicals. However, the frequent application of chemicals presents long-term risks to human health and the environment [4]. There is also evidence that pathogens have developed resistance to chemicals [5]. To avoid these undesirable consequences, current research efforts are heavily focused on the development of less hazardous practices and/or methods for crop protection.

Biological control using beneficial microorganisms is one of the most environmentally sound and economically viable ways to manage plant diseases. These microorganisms aid plants by promoting growth and suppress phytopathogens due to their antagonistic activity [6]. In the rhizosphere, plant growth-promoting rhizobacteria (PGPR) are important bacterial groups that respond to soil-borne diseases and function as biocontrol agents [7]. This activity is largely due to their production of antimicrobial compounds and hydrolytic enzymes, which makes them an excellent, environmentally friendly, and sustainable alternative to indiscriminate chemical treatment for controlling plant pathogens. The development of reliable microbial treatments to promote plant growth requires, however, the identification of new beneficial bacterial strains with high antagonistic activity towards phytopathogens. Several mechanisms have been suggested to explain the antagonistic effects of beneficial microorganisms on phytopathogens, including competition for nutrients, minerals, and colonization sites [8], inhibition of the pathogens via secreted toxins, antibiotics and biosurfactants [9], and parasitism based on the production of hydrolytic enzymes that degrade pathogens’ cell walls [10].

*Pseudomonas* spp. (Pseudomonadaceae) is one of the most abundant genera of beneficial rhizobacteria among the large and heterogeneous bacterial populations in the rhizosphere and has, therefore, attracted growing attention as a source of potential biological control agents [11,12]. Except for some human pathogenic strains [13], the species of *P. aeruginosa* is a versatile and ubiquitous bacterium that has been recognized as an active antagonist of several bacterial and fungal plant pathogens, and has potential practical applications in agricultural systems [14]. This species can produce secondary metabolites such as indole acetic acid (IAA) and siderophores, and can also solubilize phosphate [15]. Research on its interactions with plant-infecting fungi such as *Alternaria*, *Rhizoctonia*, and *Sclerotium* and oomycetes such as *Pythium* and *Phytophthora* have demonstrated that its production of phenazines plays an important role in controlling these pathogens [16]. In addition, *P. aeruginosa* isolates have shown antagonistic activity against *Pythium myriotylum* and *Phytophthora capsici* in black pepper [17], *Sclerotium rolfsii* in cucumber [18], *Xanthomonas* sp. infecting various crops [19], *P. myriotylum* in ginger [20,21], *Colletotrichum gloeosporioides* in chili [22], *Ralstonia solanacearum* in tomato [23], and *Fusarium oxysporum* in cotton [24] and wheat [25].

The present study was conducted to shed light on the plant growth-promoting activity of the recently isolated *P. aeruginosa* strain FG106 and its antagonistic activity against *A. alternata*, *R. solani*, *X*. *euvesicatoria* pv. *perforans*, *C. michiganensis* subsp. *michianensis*, *P. infestans*, *P. colocasiae*, and *B. cinerea*. The specific objectives of this work were to (1) determine the effects of the FG106 strain on growth and development of the above-mentioned plant pathogens, (2) evaluate the biochemical and enzymatic activities of *P. aeruginosa* FG106, (3) investigate the strain’s effects on tomato germination and growth, (4) characterize the interactions between FG106 and selected bacterial and fungal and oomycete pathogens and diseases under greenhouse conditions, and (5) sequence the strain’s genome to identify the genetic origin of its plant growth-promoting activity and phytopathogen antagonism. 

## 2. Materials and Methods

### 2.1. Isolation of Biocontrol Bacteria 

For isolation of bacteria, root samples were randomly collected from healthy tomato roots grown in different parts of Khorasan Razavi, a province of Iran. Samples were immediately put into a paper bag, transferred to the laboratory, and stored at 4 °C. Roots were washed with tap water, then surface sterilized for 2 min in 70% ethanol and 5 min in 3% sodium hypochlorite solution followed by three washes with sterile water. The water from the third wash was used as a control sample. The roots were crushed and shaken for 20 min on a shaker at 220 rpm, then used to generate a serial dilution up to 10^−5^. Pseudomonad strains were isolated using the spread plate technique on King’s B medium agar. After assessment for antagonistic interactions with *R. solani*, the isolate showing the strongest antifungal effect, i.e., FG106, was selected for further screening and stored in Luria-Bertani (LB) broth (Duchefa Biochemie, Haarlem, The Netherlands) containing 30% glycerol at −80 °C [26,27].

### 2.2. In Vitro Antagonistic Activity

Strain FG106 was tested against the following plant pathogens to evaluate its antagonistic activity: *A. alternata* and *R. solani* AG4-HG II, *P. infestans* 88069, *P. colocasiae* 7290 [28] and *Botrytis cinerea* B05, the Gram-positive bacterium *Clavibacter michiganensis* subsp. *michiganensis* strain PVCT156.1.1, and the Gram-negative bacterium *Xanthomonas euvesicatoria* pv. *perforans* strain NCPPB4321. Before dual culture assays, the test pathogen strains were maintained on appropriate media: *R. solani* (with a growth period of 7 days) and *A. alternata* (14 days) on potato dextrose agar (PDA, Sigma-Aldrich, Germany), *P. infestans* on rye agar (14 days), and *P. colocasiae* (10 days) and *B. cinerea* (7 days) on corn meal agar (CMA, Sigma-Aldrich, Germany).

Dual culture assays were performed in Petri dishes on selective media. The FG106 strain was cultured on either side of the Petri dish, with the initial inoculation being performed 1 cm from the edge of the plate. An agar plug 5 mm in diameter containing the fungal or oomycete pathogen to be tested was then placed in the center of the plate. The resulting plates were incubated at 20 ± 2 °C until the leading edge of the fungus in a control plate containing only the pathogen without FG106 reached the edge of the plate. The antagonistic effect of the bacterial strain on the fungus/oomycete was quantified by computing its inhibition rate as a percentage using below Equation (1) [27,29]:

(1) $$\mathrm{Inhibition}\;\mathrm{rate}\;\%=\frac{\mathrm{RC}-\mathrm{RI}}{\mathrm{RC}}\times 100\;\;$$

where RI and RC are the minimum distance between the center and the margin of the fungus in the treatment plates and the distance between the center and the margin of the fungus in the control, respectively. This experiment was performed using a completely randomized design with at least 6 replicates per pathogen.

The antagonistic activity of FG106 against the plant pathogenic bacteria *C. m.* subsp. *michiganensis* and *X. e.* pv. *perforans* was tested on LB agar plates (6 cm). Suspensions of one pathogenic bacterium in sterile distilled water (OD600 = 0.1) obtained from overnight cultures in nutrient broth (NB) were swabbed uniformly across an LB agar plate, which was then dried and spot-inoculated with an FG106 isolate. The plates were then incubated at 28 °C for 1–5 days. The efficiency of *P. aeruginosa* in suppressing the pathogen’s radial growth was determined as described previously [30]. The antibacterial activity was scored on an arbitrary scale ranging from 0 to 3 based on the scale of growth inhibition around the FG106 colony, where 0 means no halo, 1 refers to halo less than 5 mm in diameter, 2 indicates a 5–10 mm halo, and 3 is a halo larger than 10 mm. Plates inoculated with the pathogens alone were used as positive controls. Three independent replicates were performed for each tested pathogen and control [31].

### 2.3. Preliminary Identification of Strain FG106

The DNA of the FG106 strain was isolated using a Quick-DNA Fungal/Bacterial Microprep Kit according to the manufacturer’s recommendations (Zymo Research, Irvine, CA, USA). DNA yield and integrity were measured using a NanoDrop micro photometer (NanoDrop Technologies, South San Francisco, CA, USA) and by agarose gel electrophoresis, respectively. The 16S rRNA gene of FG106 was PCR-amplified using the primer pairs 27F (5′-AGAGTTTGATCMTGGCTCAG-3′) and 907R (5′-CCGTCAATTCMTTTRAGTTT-3′) [32]. The PCR was performed using 10 ng of FG106 DNA with the following temperature parameters: initial denaturation at 94 °C for 3 min, followed by 35 cycles of 94 °C for 45 s, 50 °C for 30 s, and 72 °C for 30 s, with a final extension step at 72 °C for 5 min. The PCR products were purified using the Qiagen PCR purification kit (Qiagen, UK). The purified PCR products were sequenced for species identification at the Eurofins sequencing facility (Germany). SnapGene software was used (SnapGene, San Diego, CA, USA) to manually analyze and edit the nucleotide sequence obtained from the sequencing platform. The resulting sequence containing the 16S region was searched for matching hits against the National Center for Biotechnology Information (NCBI) GenBank non-redundant nucleotide database (BLASTn) [33]. Search hits to sequence from records in the database were analyzed for sequence coverage and identity, and the best matched NCBI accession was recorded. 

### 2.4. Production of Volatile and Non-Volatile Metabolites 

The antibacterial activity of volatile compounds (VOCs) from the FG106 strain was investigated as described by Nishino [34]. Briefly, strain FG106 was cultured on a plate with tryptic soy agar (TSA, Sigma-Aldrich, Taufkirchen, Germany) medium, and a 5 mm mycelial plug of one pathogen was separately placed at the center of another plate on specific culture medium. The Petri dishes containing the bacterium and pathogen were then placed face to face, sealed with parafilm, and incubated at 28 °C. A plate containing bacterium-free TSA medium was used as a control.

To investigate the biocontrol effects of non-volatile compounds (NVOCs), the FG106 strain was cultured in tubes containing tryptic soy broth (TSB, Sigma-Aldrich, Taufkirchen, Germany) medium on a shaking incubator (Thermo Scientific, Waltham, MA, USA) for 16 h. The bacterial suspension was then centrifuged at 4200 rpm for 15 min. Two mL of the supernatant was filtered through a 0.22-micron MilliPore (MP) filter, (Sarstedt, Nümbrecht, Germany) supplemented with 18 mL of an appropriate medium in a 9:1 ratio, and placed in a Petri dish. A 5 mm mycelium disk was then placed in the center of the prepared medium. The resulting Petri dishes were maintained at a temperature of 20 °C. The radial growth of the fungal/oomycete colony was measured, and its inhibition percentage was calculated using the previously reported equation [35,36]. At least six replicates were performed per tested pathogen.

### 2.5. Evaluation of Hydrolytic Enzyme Activity of FG106

The production of cellulase enzyme activity by the FG106 strain was assessed as previously described [37,38], along with protease [38,39], chitinase [40,41], pectinase [42], amylase [27,43], and lipase production [44].

### 2.6. Investigation of Plant Growth Factors

Inorganic phosphate solubilization by strain FG106 was assessed on Pikovskaya agar (Sigma-Aldrich, Germany) with incubation for 7 days at 28 °C [45], potassium solubilization on Alexandrov medium (Sigma-Aldrich, Taufkirchen, Germany) [46,47], and ammonia production on peptone water agar with added Nessler’s reagent (Sigma-Aldrich, Taufkirchen, Germany) [48]. Medium amended with tryptophan was used to screen for indole acetic acid (IAA) production [49]. Quantitative determination of IAA was performed using the Acuña method [50].

### 2.7. Production of Hydrogen Cyanide (HCN) and Siderophores, and Biofilm Formation

To screen for HCN production, strain FG106 was cultured on glycine-containing King’s B medium that was then pressed against filter paper impregnated with picric acid (Sigma-Aldrich, Taufkirchen, Germany) [51,52]. The siderophore assay was performed using Chrome Azurol S medium (Sigma-Aldrich, Taufkirchen, Germany) containing iron chloride [52,53]. Biofilm formation was determined in 96 -ell polystyrene microtiter plates (Nunc^™^ MicroWell^™^ 96-well and flat- bottom microplate, Thermo Fisher Scientific, Waltham, MA, USA) [54].

### 2.8. Biosurfactant Production

Sterile distilled water was placed in a Petri dish, and the surface of the dish was completely covered with sunflower oil. Ten microliters of a bacterial supernatant obtained by centrifuging a 10^7^ cfu/mL bacterial suspension at 3000 rpm for 10 min was then placed on the oil. It was concluded that the bacterium had the capacity to produce biosurfactants if a clear oil-free zone formed around the bacteria [55,56].

### 2.9. In Vitro Investigation of the Effect of Strain FG106 on Growth of Tomato Seedlings

Sterile seeds of the tomato cultivar ‘Money Maker’ were soaked in a 2 × 10^7^ cfu/mL bacterial suspension for 30 min. After drying, the seeds were transferred to solid 50% Murashige and Skoog (MS) medium (Duchefa Biochemie, Haarlem, The Netherlands). After 14 days, the length of the resulting plants was measured. Control seeds were soaked in 1× phosphate buffered saline (PBS) (PanReac AppliChem, Darmstadt, Germany) [57].

### 2.10. Biocontrol Activity Using Detached Leaf Assay (DLA)

Leaves from 3 to 4 week-old potato and strawberry plants were washed and inoculated with 20 µL of a 2 × 10^7^ cfu/mL suspension of strain FG106. After 24 h, 20 μL of a challenge pathogen suspension was added individually; the concentrations of these suspensions were 25,000 sporangia/mL for *P. infestans* or 50,000 sporangia/conidia mL for *P. colocasiea* or *B. cinerea* [58]. Disease assessment was performed 6 days post-inoculation; the leaf area exhibiting symptoms of infection was measured using the ImageJ software package [59] and compared to that in control leaves. Control leaves were treated with 20 μL 1× PBS buffer instead of a pathogen suspension. This experiment was repeated three times with 5 leaves per treatment and two spots per leaf [60,61]. 

### 2.11. Greenhouse Trials 

Three important tomato pathogens *R. solani*, *C. m.* subsp. *michiganensis*, and *X. e.* pv. *perforans* were selected for greenhouse trials to test the antagonistic potential of FG106. 

#### 2.11.1. *Rhizoctonia solani*

Tomato seeds (*L. esculentum*, Mobil) were surface sterilized for 5 min in 2% sodium hypochlorite and rinsed twice with 70% ethanol, followed by three washes with sterile distilled water. Seeds were placed in sterilized plastic pot trays containing perlite soil and then grown for 4 weeks in a growth chamber with daily watering. Roots of 4-week-old seedlings were then dipped in a 2 × 10^8^ cfu/mL suspension of strain FG106 for 20 min, while control seedlings were treated with distilled water [62]. The next day, the seedlings were inoculated with 3 g of wheat seeds colonized with *R. solani* [36,63]. Plants were kept in the greenhouse in a high humidity chamber for 12 h before and 12 h after inoculation to maintain high humidity and facilitate infection. Ten pots with one seedling per pot were used in each treatment. Disease severity was scored 15 days later using a previously reported scale [26]. The disease index (DI) was scored as described previously [64]; briefly, a score of 0 indicated no visible necrotic lesions, 1 indicated root necrosis up to 2.5 mm in length, 2 indicated necrosis 2.5–5.0 mm in length, 3 indicated necrosis larger than 5.0 mm, 4 indicated lesions covering the crown and shoots, and 5 indicated seedling damping-off. 

#### 2.11.2. Preparation of Inoculum of Bacterial Pathogens and Bacterial Antagonists 

*C. m.* subsp. *michiganensis* and *X. e.* pv. *perforans* were selected to evaluate the biocontrol activity of FG106 on tomato plants in vivo. Inocula of both pathogens and the biocontrol agent (FG106) were prepared from bacterial cells grown for 48 h on nutrient agar (NA) (Sigma-Aldrich, Taufkirchen, Germany). Single colonies were transferred into LB broth and subsequently incubated at 28 °C for 48 h in a rotary shaker at 150 revolutions/min (rpm). The bacterial cultures were then centrifuged for 15 min at 7500 rpm, and the pellets were resuspended in sterile water, after which their density was adjusted to 2 × 10^8^ cfu/mL (OD600 = 0.1), and they were used in pathogen challenge experiments with 4–5 true-leaved tomato seedlings [65,66,67].

#### 2.11.3. Plant Material and Inoculation of Bacterial Endophytes 

Three-week-old seedlings of the hybrid tomato cultivar ‘SIR ELYAN F_1_′ were obtained from a local nursery [65]. Fifteen seedlings were tested for each treatment. The trials were designed to evaluate biocontrol effect of FG106 on the formation of bacterial cankers *C. m.* subsp. *michiganensis* and bacterial spots *X. e.* pv. *perforans*. Plants were maintained in a growth chamber at 68–80% RH and 25 °C, with 16/8 h of light and darkness daily. Experiments were conducted in duplicate. The bacterial inoculations were performed by soil drenching with 20 mL of the appropriate bacterial suspensions. Tomato seedlings were harvested after 30 days. Negative controls consisted of seedlings drenched with tap water instead of the pathogen suspensions. 

#### 2.11.4. Plant Competition with Bacterial Pathogens

Inoculation of *C. m.* subsp. *michiganensis* on the plants was performed as described previously [65]. Briefly, after 7 days of treatment with the putative BCA or water (negative control), 20 mL of *C. m.* subsp. *michiganensis* solution was poured onto the soil near the stem crown. To facilitate bacterial penetration, the roots were damaged by cutting with a scalpel at three points located 2 cm from the stem crown. After 4 weeks, bacterial canker symptoms were assigned a disease index score on a scale ranging from 0 to 5, where 0 means no symptoms, 1 refers to loss of turgor and chlorosis, 2 is wilt and/or cankers > 0.5 cm in diameter in 1 or 2 leaves, 3 indicates wilt or cankers > 0.5 cm in diameter in 3 or more leaves, 4 represents fully withered plants, and 5 denotes dead plants [66]. Area under disease progress curves (AUDPCs) were generated based on weekly monitoring data for 4 weeks post-incubation [65]. 

*X. e.* pv. *perforans* was sprayed onto the abaxial and adaxial leaf surfaces of tomato seedlings using hand-trigger sprayers 3 days after treating the soil with either FG106 or water (negative control). The RH was increased to promote bacterial penetration, and the inoculated plants were pre-incubated and post-incubated for 24 h under polyethylene sheets. Ten days after pathogen inoculation, 10 tomato leaflets per plant were sampled randomly. Spots and lesions on the leaflets were counted and the leaflet area determined, and disease severity was evaluated as number of lesions/cm^2^. The leaflet area was quantified by image processing and analysis using the ImageJ software package [68,69]. The percentage reduction in disease severity caused by the presence of FG106 was calculated relative to that seen in negative controls as described previously [68]. 

### 2.12. Colonization of Tomato Seedlings by Strain FG106 

Tomato plants (Money Maker) were planted in a seedling tray and then transferred to pots after 4 weeks. During the transfer, the roots were immersed in a suspension of FG106 with a concentration of 2 × 10^8^ cfu/mL, while negative controls were immersed in water. All seedlings were kept at 25–28 °C and 60% humidity with a 14/10 h light/dark cycle at a light intensity of approximately 500 μmol m^−2^ s^−1^. 

The number of colony forming units per gram of sample was determined using TSA media supplemented with chloramphenicol (Sigma-Aldrich, Taufkirchen, Germany) with a final concentration of 100 µg mL^−1^. To evaluate the presence of bacteria in the leaves and roots, the leaf and root surfaces were surface sterilized for 5 min with 3% sodium hypochlorite, followed by three washes with sterile water. Water from the final wash was used as a negative control. Plant tissue was homogenized aseptically with a pestle and mortar, and bacterial CFUs were enumerated by dilution and plate counting as described previously [70]. Homogenized samples (1 g per replicate) of the rhizosphere, root, and leaves were added to 9 mL of 1× PBS solution, separately. Samples were then vortexed for 1 min and serially diluted at 1:10 until a 10^9^ dilution was reached. Aliquots of 100 µL were plated from each dilution onto agar plates and then incubated at 28 °C for 48 h. Untreated plants maintained under similar conditions were also subjected to this procedure to detect naturally occurring bacteria capable of growing on the TSA medium [71]. 

Bacterial populations in the rhizosphere, roots, and leaves of the plants were assessed at 15, 30, and 45 days post-inoculation (dpi) to investigate the survival of the bacteria in the plant and rhizosphere. The height, chlorophyll content, number of leaves, and fresh and dry weight of the seedlings were also recorded. Six replicates were analyzed per treatment [70,72,73].

### 2.13. Genome Sequencing and Assembly 

The *P. aeruginosa* strain FG106 was grown in 20 mL of LB medium and incubated for 18 h at 28 °C. After this period, total genomic DNA was extracted using the Nanobind CBB Big DNA Kit (Circulomics Kit, Baltimore, MD, USA) according to the manufacturer’s recommendations. DNA quality and quantity were checked by agarose gel electrophoresis and using a NanoDrop instrument (NanoDrop Technologies, South San Francisco, CA, USA). Library construction was performed using the Illumina TruSeq PCR-free kit (Thermo Scientific, Waltham, MA, USA)) with an insert size of 670 bp. The FG106 genome was sequenced at the SciLifeLab, Sweden, on a MiSeq instrument (MSC 2.5.0.5/RTA 1.18.54) with a 2 × 300 bp reads setup using ‘Version3’ chemistry. The SPAdes version 3.14.1 software package was used for sequence assembly and quality assessment. The whole genome sequencing raw data were submitted to the BioProject: ID PRJNA767521. The genome assembly data have been deposited at DDBJ/ENA/GenBank under the accession: JAJNEF010000000.

### 2.14. Predicted 16S rRNA Gene

The 16S rRNA gene sequence was predicted based on the constructed primary genome assembly using RNAmmer 1.2 4, which predicts 5s/8s, 16s/18s, and 23s/28s ribosomal RNA in full genome sequences [74]. 

### 2.15. Draft Assembly Circular Map

A circular map of genome features was constructed using the CGView tool [75]. Final draft assembly was performed using the CGView Server. The CGView Comparison Tool (CCT) package [76] was used to perform clusters of orthologous groups (COG) classifications and generate a circular plot showing DNA-vs.-DNA mappings for the FG106 strain and two reference strains: P.aeru-DSM.50071 (NZ_CP012001) and P.aeru-M18 (NC_017548). 

The COG categories were generated by searching the COG and CDD database from 2003–2014 using rpsblast (ncbi-blast-2.12.0+). The resulting data were processed using the cdd2cog.pl perl script (https://github.com/aleimba/bac-genomics-scripts/tree/master/cdd2cog (accessed on 16 November 2021)) to classify functional groups.

### 2.16. Gene Detection and Analysis of Coding Genes from FG106

The virulence factor database (VFDB) [77] was searched to retrieve bacterial VF categories for detected genes, and additional factors were added from the literature. Comparative mapping was performed by BLAST searching using eight complete and closely related genomes of *P. aeruginosa* strains, namely, P.aeru-LESB58, P.aeru-PA7, P.aeru-PAO1, P.aeru-UCBPP-PA14, P.aeru-DSM.50071, P.aeru-M18, P.aeru-PAO581, and P.aeru-L10.

### 2.17. Draft Genome Gene Prediction, Annotation and Functional Characterization

Gene prediction and annotation based on the draft genome were performed using the RAST annotation server (Rapid Annotation using Subsystem Technology (http://rast.nmpdr.org (accessed on 16 November 2021)) with the following annotation parameters: genetic code = 11, E value cut-off for selection, and pinneMetricd CDSs = 1 × 10^−20^. 

### 2.18. Secondary Metabolite Analysis

Secondary metabolite analysis was performed using antiSMASH to identify gene clusters encoding enzymes synthesizing secondary metabolites belonging to all known broad chemical classes.

### 2.19. Comparative Genomics and Phylogenomics

The average nucleotide identity (ANI) value is widely used to compare prokaryotic genome sequences and to classify and identify bacteria [78]. ANI was calculated using the nearest reference genome (*P. aeruginosa* PAO1) to the sample’s assembled genome (FG106) using Chulabs’s online Average Nucleotide Identity (ANI) calculator. The average amino acid identity (AAI) is a very robust measure of genetic and evolutionary relatedness between two strains that correlates strongly with DNA–DNA association values (the classical tool for species delineation in prokaryotes) and the genome’s mutation rate. For the phylogeny analysis based on the complete genome, we used iTOL (https://itol.embl.de (accessed on 16 November 2021)) with Dendroscope with the whole genomes of 37 *P. aeruginosa* strains (including the genome of strain FG106 and 36 other genomes), which led to constructing a phylogenetic tree based on maximum parsimony. This analysis grouped strain FG106 in a clade with five other *P. aeruginosa* strains (RTE4, RP73, SCV20265, C3719, DK2).

### 2.20. Statistical Analysis 

Data from the experiments on the antagonistic effects of FG106, the tomato growth experiments, chlorophyll content measurements, and the bioassays were analyzed using SAS statistical software version 9.2 (SAS Institute, 2009). One-way ANOVA was used to compare parameters with at least three independent groups. Tukey’s test with a significance threshold of *p* < 0.05 was used to compare differences between paired means. For parameters with two independent groups, differences between means were evaluated using the *t*-test.

## 3. Results

### 3.1. Isolation and Molecular Identification of Strain FG106 

The bacterial strain FG106 was isolated from the roots of healthy tomato plants in Khorasan Razavi province. After purification, the strain was cultured on King’s B medium and kept at 25 °C for 2 days. Bacterial colonies produced pyoverdine pigments in this medium upon exposure to UV light. Microscopic observations showed that FG106 has a rod shape. Sanger sequencing of a portion of the 16S rRNA gene enabled the identification of FG106 as a *Pseudomonas aeruginosa* strain based on BLASTn analysis. The sequencing data are provided in the Supplementary Materials (Table S1).

### 3.2. Antagonistic Activity Assay of FG106 Strain

**Figure 1:**
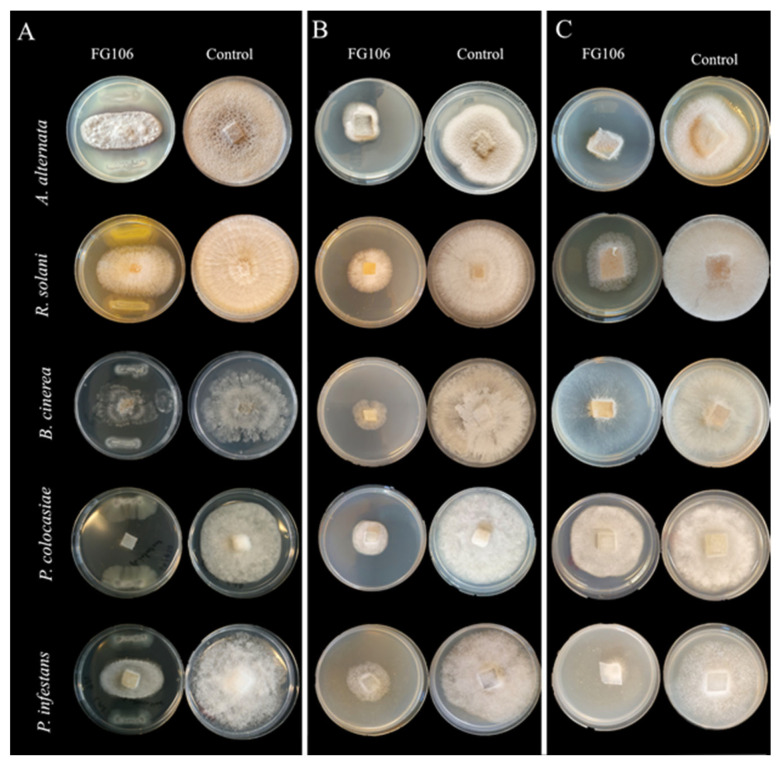
Determination of in vitro antagonistic activity of *Pseudomonas aeruginosa* strain FG106 against selected phytopathogens. (**A**) Antagonistic potential of FG106 strain in dual culture tests. (**B**) Antimicrobial effects of non-volatile metabolites produced by strain FG106. (**C**) Inhibitory effect of volatile metabolites produced by strain FG106.

**Figure 2:**
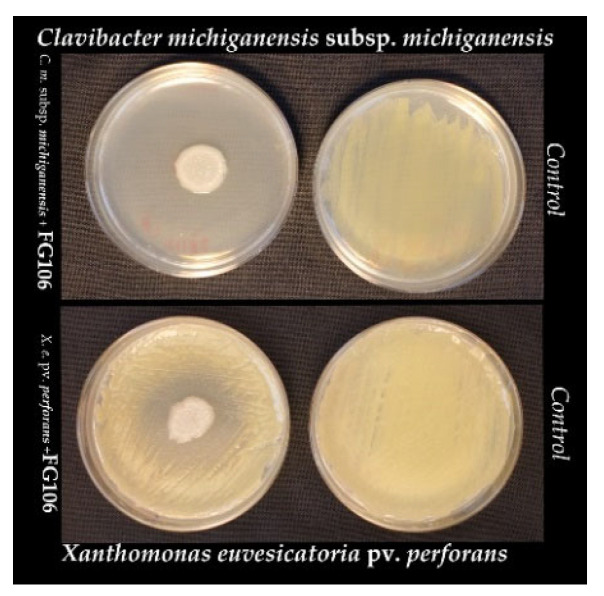
Antagonistic effects of *P. aeruginosa* strain FG106 against *Clavibacter michiganensis* subsp. *michiganensis* and *Xanthomonas euvesicatoria* pv. perforans in vitro.

In dual culture assays, FG106 inhibited the growth of all tested plant pathogens, including oomycetes, fungi (Figure 1), and bacteria (Figure 2). It achieved its highest inhibition rate of 83.4% in experiments with *P. infestans*, followed by *A. alternata* (58.9%*)*, *P. colocasiae* (56.5%), and *B. cinerea* (47.6%); the lowest inhibition rate of 44.6% was seen for *R. solani* (Table 1). The bacterium also inhibited the growth of the Gram-positive bacterium *C. michiganensis* subsp. *michiganensis* and the Gram-negative bacterium *X. euvesicatoria* pv. *perforans*; in these cases, its inhibitory activity levels were scored as 3 and 2, respectively, based on the radius of the inhibition halo (Figure 2). 

The inhibition rate of the VOCs produced by FG106 ranged from a maximum of 90.1% (*P. infestans*) to a minimum of 33% (*P. colocasiae*). The inhibition rates of its NVOCs ranged from 61.1% for *B. cinerea* and 60.9% for *P. colocasiae* to 48.3% for *A. alternata* (Table 1). 

### 3.3. Evaluation of Plant Growth Promotion and Hydrolytic Enzyme Production by the FG106 Strain

**Figure 3:**
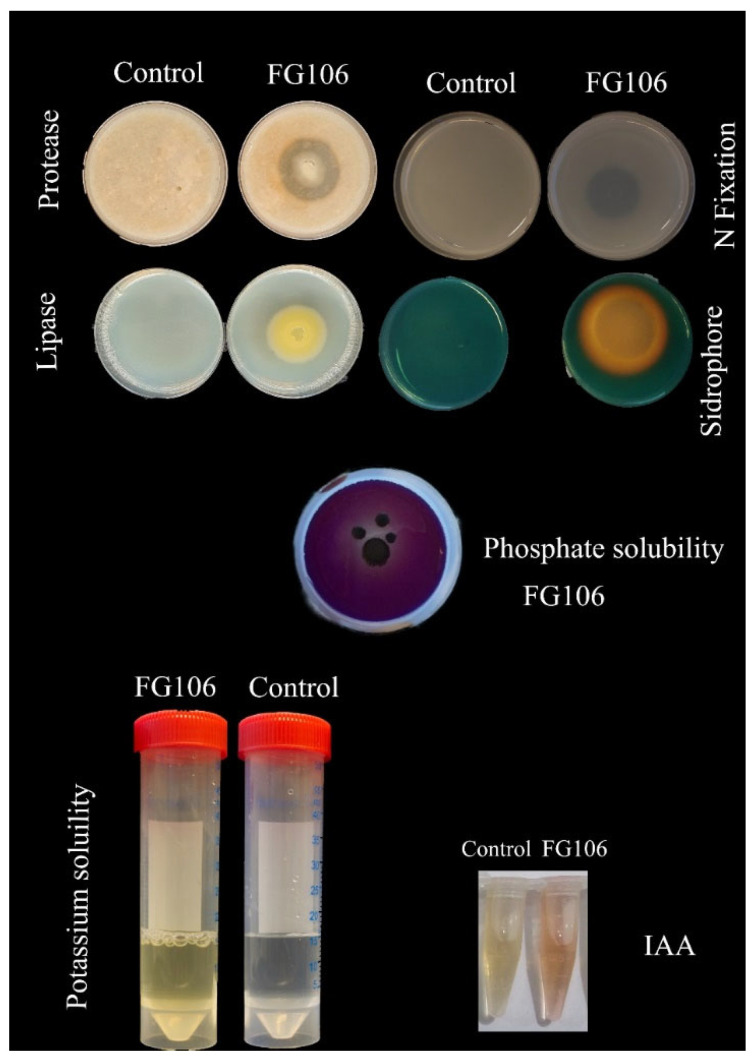
Hydrolytic enzyme activity and growth promotion factor production by the FG106 strain in experiments targeting protease and lipase production, nitrogen fixation, siderophore production, phosphate solubilization, potassium solubilization, and IAA production.

Clear halo zones with diameters of 1.9 and 0.7 cm (Table 2) were observed on peptone water agar and Pikovskaya media, respectively, indicating ammonia production and inorganic phosphate solubilization activity (Figure 3). Additionally, a change in color from blue to orange due to ferric ion transfer was observed in experiments using CAS medium, indicating that FG106 can produce siderophores. This strain also produced IAA, with a peak production of 211 µg/mL after 96 h incubation. Finally, enzyme assays showed that the strain secreted both protease and lipase enzymes, forming clear halos with diameters of 1.2 ± 0.33 and 0.2 ± 0.1 cm, respectively (Table 2). 

### 3.4. Effects of FG106 on Tomato Seedling Growth on MS Media

**Figure 4:**
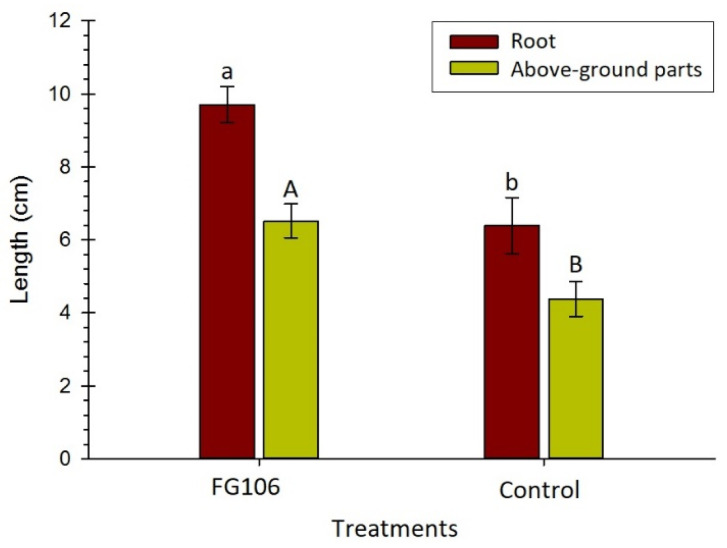
Longitudinal root growth ratios of tomato plants after bacterial inoculation with FG106 and under control conditions. Error bars represent the standard deviation (number of samples = 18); means labelled with different letters differ significantly according to Student’s *t* test at *p* < 0.01).

Experiments on tomato seedlings grown on MS media (Figure 4) revealed that treatment with the FG106 led to a significant increase in root and above-ground length (9.70 and 6.52 cm, respectively) when compared to untreated control plants (6.39 and 4.38 cm, respectively) (*t* < 0.05). In addition, treatment increased the number of lateral roots. 

### 3.5. Biocontrol Activity Using Detached Leaf Assay (DLA)

**Figure 5:**
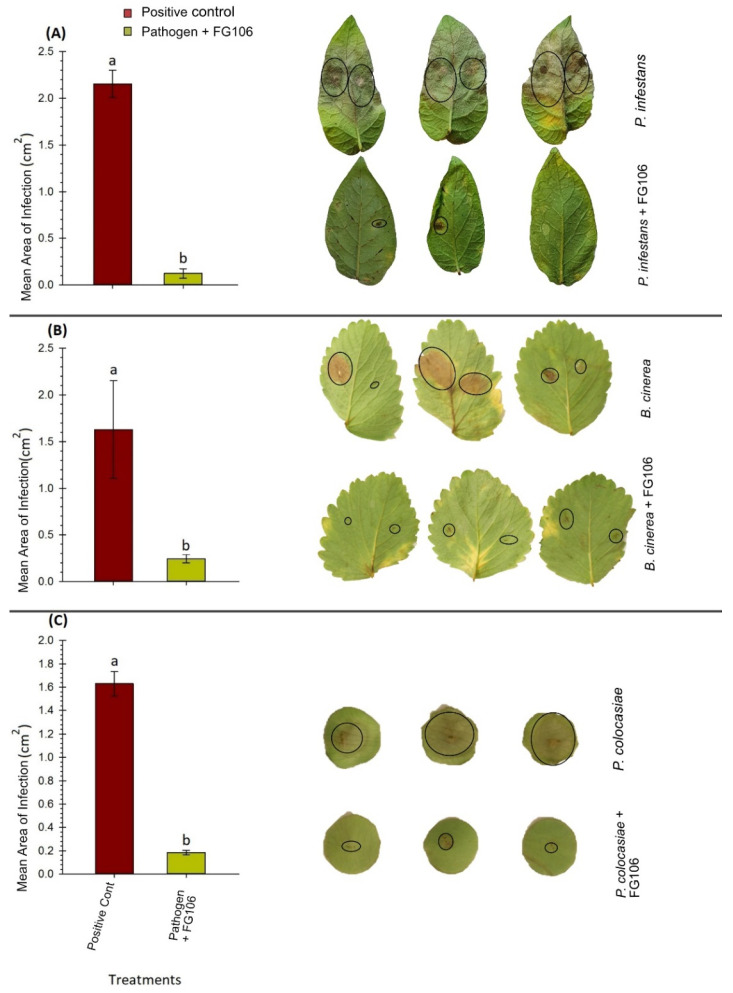
Detached leaf assay (DLA) measuring the antagonistic activity of the FG106 against (**A**) *Phytophthora infestans* on potato leaves (*n* = 16), (**B**) *Botrytis cinerea* on strawberry leaves (*n* = 12), and (**C**) *Phytophthora colocasiae* (*n* = 15) on taro leaves. In all cases, the area exhibiting symptoms was quantified using ImageJ. Data represent means ± standard deviation of 6 replicates. Means labelled with different letters differ significantly according to Student’s *t* test at *p* < 0.01).

DLA experiments targeting the interaction of FG106 with *P. infestans* on potato leaves revealed strong disease reduction, with the area of infection being reduced to 0.1 cm^2^, compared to 2.1 cm^2^ in controls. A lower level of disease inhibition was seen in strawberry leaves infected with *B. cinerea* (area of infection = 1.6 cm^2^ in controls and 0.2 cm^2^ after treatment). FG106 also exhibited antagonistic effects against a taro pathogen (*P. colocasiae*), indicating that it has a broad spectrum of inhibition (Figure 5).

### 3.6. Greenhouse Trials

#### 3.6.1. Interaction between the Prospective Biocontrol Agent (BCA) FG106 and the Fungal Pathogen *R. solani* under Greenhouse Conditions

The disease index (DI) of plants treated with *R. solani* and BCA (0.64) was significantly lower than that of plants treated with *R. solani* alone (1.23) (Table 3). In addition, FG106 treatment increased key plant parameters including fresh and dry root and shoot weights, demonstrating that this strain is a BCA with plant growth-promoting activity. 

#### 3.6.2. Interaction of FG106 with the Pathogenic Bacterium *C. m.* subsp. *michiganensis* and *X. e.* pv. *perforans* under Greenhouse Conditions

Disease progression was monitored for 4 weeks in tomato seedlings treated with the FG106 strain and challenged with the pathogenic bacteria *C. m.* subsp. *michiganensis*, and in positive controls treated with the pathogen but not FG106 (Table 4). The first symptoms of bacterial canker caused by *C. m.* subsp. *michiganensis* were leaflet formation and unilateral wilting, which were seen only in control plants starting at 14 days post-inoculation (dpi). General wilting symptoms were seen in all treatments at 21 dpi. The severity of the wilting increased gradually over time. By week 5 post-inoculation, the disease index of the plants treated with FG106 and *C. m.* subsp. *michiganensis* (2.57) differed significantly (*p* < 0.05) from that of the positive controls (3.67). Additionally, mortality was observed in the positive controls but not in the FG106 + *C. m.* subsp. *michiganensis* plants. 

The antagonistic effects of FG106 on the pathogenic bacterium *X. e.* pv. *perforans* were also investigated. Lesion formation was observed on the leaves of positive control (*X. e.* pv. *perforans*) plants from 6 dpi and expanded chlorotic spots that had become necrotic were visible at 10 dpi. Endophyte-treated plants had significantly fewer spots than control plants; disease severity calculations yielded 11.7 spots/cm^2^ for positive controls compared to only 3.09 cm^2^ for plants treated with FG106 (Table 4).

### 3.7. The Rate of Colonization of FG106 in Tomato Seedlings

**Figure 6:**
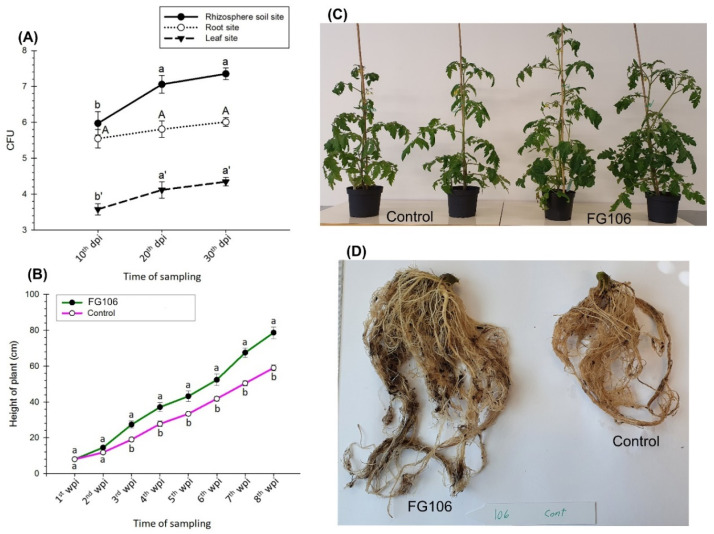
Colony-forming units (CFUs) of *P. aeruginosa* strain FG106 in the rhizosphere, roots, and leaves of tomato seedlings up to 45 days post-inoculation (**A**) and its effects on seedling height (**B**), growth of above-ground parts (**C**), and root growth (**D**). Data represent means ± SD of six replicates. Means labelled with different letters differ significantly according to Student’s *t* test at *p* < 0.01.

Compared to plants treated with just water, plants treated with a suspension of the bacterial strain FG106 showed significantly higher growth 8 weeks post-treatment. The root systems of plants inoculated with the FG106 suspension also showed significantly increased growth and development (Figure 6D), and inoculation with the endophyte increased the size of the bacterial population in the rhizosphere significantly more than in the roots and leaves at 15, 30, and 45 dpi (*p* < 0.01). This indicates efficient colonization of the rhizosphere (Figure 6A). 

The treated plants also displayed significantly increased (*p* < 0.05) fresh and dry root weight (17.1 g vs. 2.9 g for negative controls) and weight of above-ground parts (173.2 g vs. 18.3 g). Furthermore, the chlorophyll content in leaves of treated plants at the end of the experiment was approximately 21% higher than in leaves of untreated controls (Table 5). In contrast, the mean number of leaves in FG106-treated seedlings (12) was significantly (*p* < 0.05) lower than in controls (17).

Figure 6B,C shows the effect of FG106 on the height of tomato plants during the first 8 weeks after inoculation. Except for the first and second weeks, significant differences were observed between treated and untreated control plants, with the treated plants being 25% taller at the end of the eighth week.

### 3.8. Genome Sequencing and Assembly

The FG106 genome has a size of 6,283,027 bp, with a GC content of 66.55%, 5941 protein-coding genes, and 86 tRNAs. Gene ontology analysis revealed the presence of many genes related to secondary metabolism and plant growth-promoting activity.

The total number of raw reads was 7,255,218, giving 6,283,027 high-quality reads after trimming. Draft assemblies were based on 6,281,727 reads with a mean length of 201 bp, resulting in 31 total sequences with a minimum sequence length of 219 bp and a maximum sequence length of 1,343,657 bp. Among the 6004 predicted genes, 5941 were identified as protein-coding genes and 63 as protein non-coding genes. Of the coding genes, 5035 were assigned to characterized proteins, while the other 906 were designated as hypothetical proteins (Table 6). The size of the predicted 16 S rRNA gene sequence was 1531 bp.

### 3.9. Draft Assembly Circular Map

**Figure 7:**
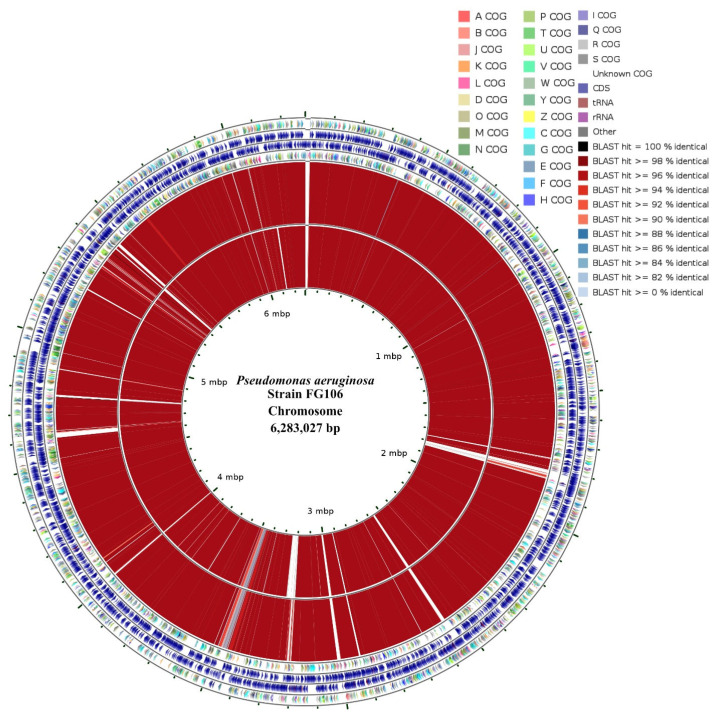
Circularized genome map of *P. aeruginosa* strain FG106 showing COG and genome comparisons and DNA-vs.-DNA comparisons. From the outside to the center, the circularized sequence shows genes on the forward strand (colored by COG categories) and genes on the reverse strand (colored by COG categories). The DNA map was generated using BLAST hits obtained by comparing the FG106 (101) strain to two reference strains: P.aeru-DSM.50071 (NZ_CP012001) and P.aeru-M18 (NC_017548), indicated in the two inner circles.

Clusters of orthologous groups (COG) classifications and a circular DNA-vs.-DNA comparative plot comparing the FG106 genome to two reference genomes based on predicted genes are presented in Figure 7. The classification of functional groups and the number of genes associated with COG (clusters of orthologous groups) are summarized in Table 7. 

### 3.10. Draft Genome Gene Prediction, Annotation, and Functional Characterization

**Figure 8:**
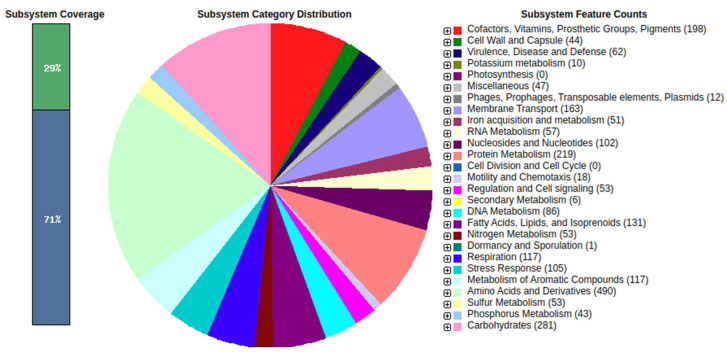
The draft genome was subjected to gene prediction and annotation using the RAST annotation server (Rapid Annotation using Subsystem Technology, http:/rast.nmpdr.org/ (accessed on 16 November 2021)).

RAST analysis for genome annotation revealed the presence of growth promotion genes involved in phosphorus, nitrogen, and potassium metabolism in the genome. In addition, the analysis predicted 51 genes belonging to the iron acquisition and metabolism category. The category with the greatest number of predicted genes was amino acid metabolism, with 490 genes (Figure 8). Additionally present were genes encoding antimicrobial compounds, siderophores, quorum sensing signals, and secretory systems that are important contributors to biological control [79]. Due to our interest in antimicrobial and antifungal compounds, the presence of genes associated with secondary metabolite production was also investigated (Table 8), revealing the presence of enzymes linked to production of antimicrobial compounds such as phenazine and HCN. However, the FG106 genome lacks genes associated with the biosynthesis of pyrrolnitrin and 2,4-diacetylphloroglucinol (DAPG). Genes encoding enzymes responsible for the synthesis of siderophores such as pyoverdine and pyochelin were detected, along with two genes linked to type IV pili synthesis, pili twitching motility related proteins, type III secretion system (TTSS) proteins, and effector proteins including avrE1, avrD1, avrB4-2, avrB4-1, avrB3, and avrB2. Some of these effectors are known to contribute to symbiosis with host plants through interactions with T3SS-secreted proteins known as ‘nodulation outer proteins’ (Nops) [80,81,82]. We also identified type VI secretion system (T6SS) clusters such as Hcp secretion island-1 encoded type VI secretion system (H-T6SS). Several studies have shown that T6SS promotes antagonistic activity against a wide range of competitor pathogens [83,84]. The FG106 genome also encodes gene clusters for the biosynthesis of ACC deaminase, and IAA, proteases, and growth-promoting factors that mediate K and P solubilization and ammonium production (Table 8). A gene comparative list is available in Supplementary Table S1.

### 3.11. Secondary Metabolites

**Figure 9:**
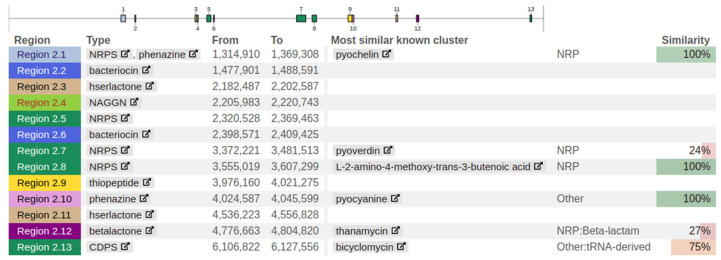
Location of secondary metabolite biosynthesis gene clusters in the FG106 genome.

The secondary metabolism of bacteria is a rich source of bioactive compounds of potential pharmaceutical value. Interestingly, the genes encoding the biosynthetic pathways responsible for secondary metabolite production are frequently spatially clustered together at specific locations in the chromosome; such collections of genes are referred to as secondary metabolite biosynthesis gene clusters. Thirteen metabolite biosynthesis pathways were identified by locating their gene clusters (Figure 9).

### 3.12. Comparative Genomics and Phylogenomics

**Figure 10:**
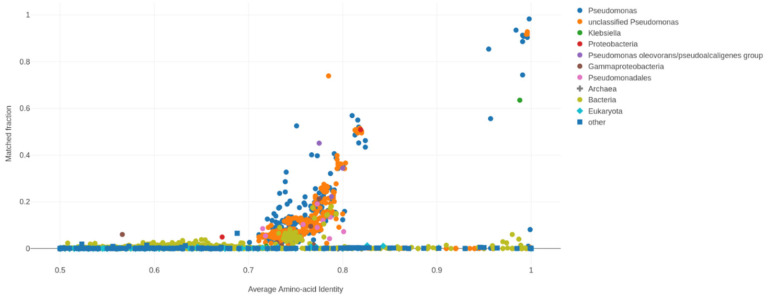
Average amino acid identity (AAI) values and fractional matches between various microorganisms including the FG106 strain.

An ANI above 95% between two genomes indicates that they belong to the same species. Table 9 presents the results of a comparative genomic analysis of FG106 and PAO1, for which the orthologous ANI value is 99.41%. The average amino acid identity (AAI) of these two genomes and a comparison of the mutation rate for the *Pseudomonas* genome to that for non-*Pseudomonas* genomes is presented in Figure 10; the match fraction for the FG106 genome and the reference genome was 0.983 (Table 10).

**Figure 11:**
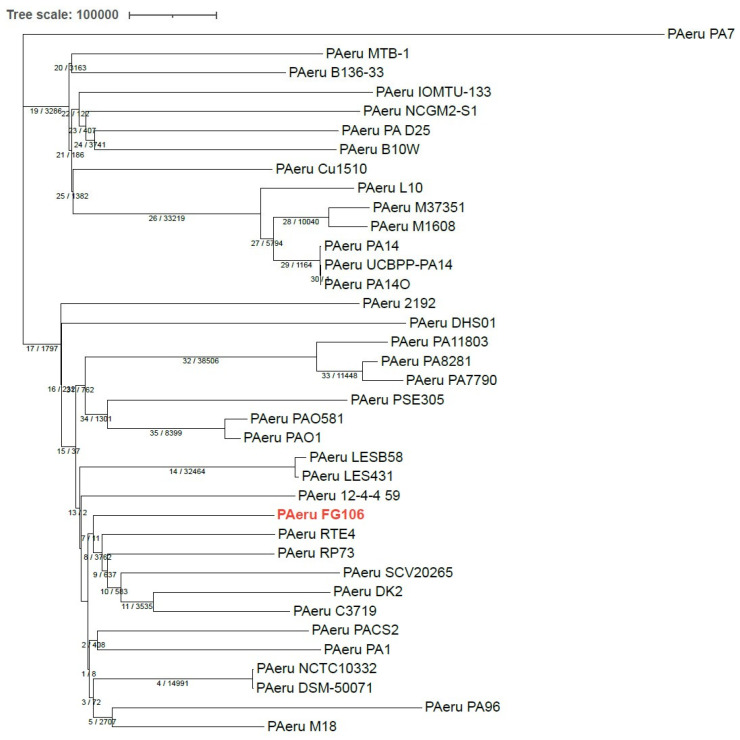
A neighbor joining (NJ) tree generated based on the number of core single nucleotide polymorphism (SNP) allele differences between sequences in the FG106 genome and 36 other *P. aeruginosa* strains. The tree was constructed using iTOL (https://itol.embl.de/ (accessed on 16 November 2021)) with Dendroscope. *P. aeruginosa* strain FG106 is written in red color. Numbers in the tree are node numbers (on the left) and numbers of SNP alleles (on the right).

A neighbor-joining (NJ) phylogenetic analysis based on the number of SNP allele differences between sequences of core SNPs from 37 *P. aeruginosa* strains showed that FG106 is closely aligned with the M18 strain (Figure 11). Wu et al. [79] found that this strain is a biocontrol agent active against a broad spectrum of pathogenic fungi and bacteria.

## 4. Discussion

A strain of *Pseudomonas* spp. designated FG106 was isolated from the rhizosphere of healthy tomato plants. Phenotypic and phylogenetic analyses revealed that this strain belongs to *P*. *aeruginosa*. In particular, *P. aeruginosa* has many interesting features that can be agriculturally valuable, mainly due to its robustness, ability to compete with pathogens, and capacity to produce secondary metabolites that inhibit pathogen growth. Accordingly, its strains are potential biocontrol agents. 

The new strain’s ability to control the pathogenicity of several pathogens was evaluated in vitro and in vivo, revealing that strain FG106 can solubilize phosphate and thus promote lateral root development and mineral absorption by the host plant [85]. It also produced siderophores, which increase plant tissue growth and root length while also increasing pathogen resistance by solubilizing otherwise insoluble iron [86]. Siderophore production by FG106 also contributes to its ability to compete effectively with pathogens for scarce iron [87], enabling FG106 to efficiently occupy an ecological niche in the rhizosphere. Another important bioactive compound produced by this strain is IAA, which increases root length. Research demonstrated that *Pseudomonas* strains produce higher levels of IAA than other beneficial bacteria [88]. The impact of this compound on the host plant is due to its status as a key phytohormone that regulates plant growth, nutrient uptake, and activation of defense responses [89]. It also plays important roles in the formation of root tissues; changes in auxin levels correlate with changes in root growth [90]. Another important quality of FG106 is its ability to produce ammonia, which facilitates the absorption of nitrate and ammonium by the host plant. Furthermore, it can produce hydrogen cyanide (HCN) and form biofilms, enabling it to effectively limit the growth of phytopathogens. Finally, FG106 secretes active protease and lipase enzymes into the culture medium, which further inhibited the growth of the tested phytopathogens. Ahmadzadeh and Sharifi-Tehrani [91] have shown that these enzymes contribute to the degradation of pathogens’ cell walls. Similarly, Zaiha et al. [92] showed that the production of proteases, chitinases, and 1-3-gluconases by beneficial strains contributes to plant resistance to the pathogen *R. solani*. 

The inhibitory effect of strain FG106 differed between the tested pathogens in vitro. Its antagonistic activity was highest against *C. m.* subsp. *michiganensis* and *P. infestans*, followed by *A. alternata*, *P. colocasiae*, *X. e.* pv. *perforans*, *B. cinerea* and *R. solani*. Conversely, Chandra et al. [14] found that *P*. *aeruginosa* was most successful at inhibiting *A. alternata* and that it limited the pathogen’s growth by inhibiting mycelial growth, radial growth, and spore germination.

Inoculation with the strain FG106 had a significant effect on germination of tomato seeds under greenhouse conditions. Previous research reports indicated that other strains of this bacterial species, such as CQ-4, stimulate seed growth and plant germination on tomato [93]. We also found that the biomass of FG106 treatment in the rhizosphere was higher than in the roots and leaves. Treatment of plants with this strain led to increases in seedling height and in the fresh and dry weight of shoots and roots. Furthermore, chlorophyll levels increased significantly in seedlings treated with FG106, probably due to an increase in their ability to absorb micronutrients. Iron is an essential micronutrient for plants that is involved in several processes including chlorophyll synthesis, maintenance of chloroplast structure, and photosynthesis. All of these processes increase plant growth and phytopathogen resistance. The capacity of FG106 to control pathogens was also tested in vivo, revealing that it successfully controlled *P. infestans* in detached leaf assays and reduced the appearance and spread of disease symptoms. Although gray mold colonized the leaf surface, the size of the disease spots was smaller than in positive control plants not treated with FG106.

We sequenced the genome of *P. aeruginosa* strain FG106, which was found to be 62,081,727 bp in length and to contain 5941 protein-coding genes along with 63 RNA-only genes. Comparative analysis revealed that the closest hit for strain FG106 is *P. aeruginosa* strain DSM 50,071; these two strains exhibited 99.93% sequence identity in the 16S rRNA gene. A variety of genes associated with anti-pathogen activity including putative TTSS and T6SS genes as well as genes apparently involved in siderophore biosynthesis were identified. In addition, the genome contains gene collections associated with the production of volatile compounds, adhesion proteins, and enzymes including proteases and ACC deaminase. These results indicate that FG106 can produce cell wall-degrading enzymes and plant growth factors.

Further functional research and comparative genomic analyses will be necessary to fully evaluate the potential of biocontrol strategies based on endophytism. However, our results are consistent with other studies showing that *P. aeruginosa* strains can improve the growth of inoculated plants and protect against various pathogens [94,95].

## 5. Conclusions

Our study revealed that *P. aeruginosa* strain FG106, a Pseudomonadales belonging to the Gammaproteobacteria that was originally isolated from the roots of healthy nursery tomato plants, produces a wide range of biologically active metabolites and is a potentially valuable biocontrol agent. Our findings suggest that biological control using this strain could facilitate the integrated and sustainable management of several fungal and bacterial pathogens on tomato, potato, taro, and strawberry crops. To develop a sustainable and effective pathogen control method, we believe that multiple control strategies including fertilization, resistant cultivars, and the use of biocontrol agents will have to be applied in combination. Future research should, therefore, investigate the ability of this bacterial strain to control other plant pathogens and the mechanisms underpinning this control. 

## Figures and Tables

**Table 1:** Rates of phytopathogen inhibition by *Pseudomonas aeruginosa* strain FG106 in dual culture experiments and by its volatile and non-volatile metabolites.

Pathogen	Dual Culture Assay	Volatile Assay	Supernatant Assay (Non-Volatile)
*A. alternata*	58.9 ± 1.4 b	68.4 ± 2.2 b	48.3 ± 2.4 d
*B. cinerea*	47.6 ± 2.3 c	63.6 ± 1.4 b	61.1 ± 3.1 a
*P. colocasiae*	56.5 ± 1.5 b	33.0 ± 2.6 d	60.9 ± 0.4 a
*P. infestans*	83.4 ± 1.6 a	90.1 ± 0.4 a	56.6 ± 1.2 b
*R. solani*	44.6 ± 1.1 c	57.5 ± 2.7 c	53.7 ± 0.8 c

Data represent means ± standard deviations of six replicates. Means labelled with different letters are significantly different according to Student’s *t* test at *p* < 0.01.

**Table 2:** Plant growth promotion factors and hydrolytic enzyme production by the endophytic FG106 strain.

Characteristics	Activity	Activity Rate
Ammonia production	+	1.9 ± 0.14 cm
Phosphate solubilization	+	0.7 ± 0.1 cm
Siderophore production	+	1.01 ± 0.21 cm
Biofilm production	+	0.39 ± 0.02
Potassium solubilization	+	0.8 ± 0.08 mg/L^−1^
IAA production	+	211 (µg/mL)
HCN production	+	
Biosurfactant production	+	
Chitinase production	−	
Amylase production	−	
Cellulase production	−	
Lipase production	+	0.2 ± 0.1 cm
Pectinase production	−	
Protease production	+	1.2 ± 0.33 cm

In column 2, − indicates no activity (no halo), + indicates activity (clear halo). Data represent means ± standard deviation of six replicates.

**Table 3:** Effects of *P. aeruginosa* strain FG106 on disease index and growth parameters of tomato plants under control and biotic stress caused by *R. solani* exposure.

Parameters	FG106	*R. solani*	FG106 + *R. solani*	Control (Water)
Plant height (cm)	18.5 ± 0.3 c	16.5 ± 0.2 d	19.0 ± 0.3 b	19.5 ± 1.1 a
Fresh weight of root (g)	0.69 ± 0.02 a	0.39 ± 0.00 c	0.47 ± 0.03 b	0.50 ± 0.00 b
Fresh weight of shoot (g)	5.1 ± 0.04 a	3.43 ± 0.06 d	4.24 ± 0.02 b	4.10 ± 0.03 c
Dry weight of root (g)	0.055 ± 0.002 a	0.038 ± 0.001 c	0.050 ± 0.001 b	0.048 ± 0.002 b
Dry weight of shoot (g)	0.57 ± 0.02 a	0.30 ± 0.00 d	0.51 ± 0.00 b	0.46 ± 0.02 c
Disease Index	-	1.23 ± 0.10 a	0.64 ± 0.09 b	-

Data represent means ± standard deviation of six replicates. Means followed by different letters differ significantly according to Tukey’s test at *p* < 0.01).

**Table 4:** Disease index, dead plant percentage, number of spots per cm^2^ leaf area, and reduction percentages for tomato seedlings challenged with the pathogen *C. m.* subsp. *michiganensis* and *X. e.* pv. *perforans* with and without simultaneous treatment with FG106.

	*C. m.* subsp. *michiganensis*			*X. e.* pv. *perforans*	
Treatment	DI 30 dpi	Dead Plants (%)	AUDPC	Number of Spots/Leaf Area cm^2^	Reduction (%)
FG106 + Pathogen	2.57 a	0.00%	29.92 a	3.09 a	45.1
Positive control	3.67 b	71.42%	36.92 b	7.11 b	

DI 30 dpi: disease index based on a 0–5-point scale evaluated 4 weeks post-inoculation. AUDPC, area under the disease progress curve. Disease severity was recorded as number of spots/cm^2^ leaf area assessed 10 days post- *X. e*. pv. *perforans* inoculation. Percentage reductions in lesion numbers per unit leaf area were evaluated relative to positive controls. Means followed by different letters are significantly different (Student’s *t* test, *p* < 0.01).

**Table 5:** Effects of *P. aeruginosa* strain FG106 on root and stem weight, number of leaves, and chlorophyll content of tomato plants under greenhouse conditions.

Treatment	Number of Leaves	Weight of Stem (gr)		Weight of Root (gr)		Chlorophyll Content
		Fresh Weight	Dry Weight	Fresh Weight	Dry Weight	
Control	12.0 ± 1.1 b	101.3 ± 5.7 b	11.3 ± 0.8 b	8.8 ± 0.4 b	1.7 ± 0.1 b	18.2 ± 0.4 b
FG106	17.0 ± 1.1 a	173.2 ± 5.8 a	18.3 ± 0.9 a	17.1 ± 0.6 a	2.9 ± 0.2 a	23.2 ± 0.5 a

Data represent means ± standard deviations of six replicates. The means followed by different letters are significantly different according to Student’s *t* test at *p* < 0.01.

**Table 6:** General features of the *P. aeruginosa* strain FG106 genome.

Genome Statistics	Genome (Total)
Attribute	Value
Genome size (bp)	6,283,027
Number of genes predicted	6004
DNA coding region (bp)	5,682,531
DNA G + C content (bp)	66.55%
DNA scaffolds	2
Total genes prediction	6004
Protein-coding genes	5941
Protein non-coding genes	63
Characterized proteins	5035
Hypothetical proteins	906
rRNA genes	5
tRNA genes	58
Size of predicted 16 S rRNA gene	1531
Protein with pathway annotations	1299
Classifier predicted regions	3
CRISPR-array	4
CRISPR-repeats	53
CRISPR-spacer	49
Protein-coding genes assigned to COGs	4792

**Table 7:** Functional group classification based on COG analysis. Several genes associated with COG functional categories were identified.

Code	Description	Value
A	RNA processing and modification	2
B	Chromatin structure and dynamics	4
C	Energy production and conversion	326
D	Cell cycle control, cell division, chromosome partitioning	40
E	Amino acid transport and metabolism	520
F	Nucleotide transport and metabolism	107
G	Carbohydrate transport and metabolism	241
H	Coenzyme transport and metabolism	207
I	Lipid transport and metabolism	238
J	Translation, ribosomal structure, and biogenesis	207
K	Transcription	497
L	Replication, recombination, and repair	140
M	Cell wall/membrane/envelope biogenesis	272
N	Cell motility	158
O	Posttranslational modification, protein turnover, chaperones	200
P	Inorganic ion transport and metabolism	317
Q	Secondary metabolite biosynthesis, transport, and catabolism	173
R	General function prediction only	666
S	Function unknown	535
T	Signal transduction mechanisms	341
U	Intracellular trafficking, secretion, and vesicular transport	180
V	Defense mechanisms	73
W	Extracellular structures	0
Y	Nuclear structure	0
Z	Cytoskeleton	0

**Table 8:** Genes associated with secondary metabolite production detected in the analyzed sequences of FG106.

Class	Genes	FG106
Adherence	Type IV pili biosynthesis	+
	Type IV pili twitching motility related proteins	+
	LPS O-antigen (*P. aeruginosa*)	+
	Flagella	+
Antimicrobial compounds	Phenazine biosynthesis	+
	pyrrolnitrin	-
	2,4-diacetylphloroglucinol (DAPG)	-
	Hydrogen cyanide (HCN)	+
Antiphagocytosis	Alginate biosynthesis	+
	Alginate regulation	+
Biosurfactant	Rhamnolipid biosynthesis	+
Enzyme	Chitinase	+
	Amylase	-
	Cellulase	-
	Lipase	+
	Pectinase	-
	Protease	+
Siderophore	Pyoverdine	+
	Pyoverdine receptors	+
	Pyochelin	+
	Pyochelin receptor	+
Protease	Elastase	+
	Alkaline protease	+
	Protease IV	+
Quorum sensing	N-(butanoyl)-L-homoserine lactone QS system	+
	N-(3-oxo-dodecanoyl)-L-homoserine lactone QS system	+
	N-(3-oxo-hexanoyl)-L-homoserine lactone QS system	-
	Acylhomoserine lactone synthase	+
Regulation	GacS/GacA two-component system	+
Secretion system	*P. syringae* TTSS effectors	-
	Hcp secretion island-1 encoded type VI secretion system (H-T6SS)	+
	*P. aeruginosa* TTSS	+
	*P. aeruginosa* TTSS translocated effectors	+
	*P. syringae* TTSS	-
	Harpins, pilus-associated proteins, and other candidate TTSS helpers	-
Toxin	Exotoxin A (ETA)	+
	Phytotoxin coronatine	-
	Phytotoxin phaseolotoxin	-
	Phytotoxin syringopeptin	-
	Phytotoxin syringomycin	-
	TccC-type insecticidal toxins	-
	Exolysin	-
Immune evasion	Capsule	+
Growth promoting factors	Biofilm	+
	Biosurfactant	+
	IAA (Indole-3-Acetic Acid)	+
	tryptophan biosynthetic and IAA synthesis	+
	Ammonium production	+
	Phosphate solubilization	+
	Potassium solubility (µg/mL)	+
	ACC deaminase activity	+

**Table 9:** Average nucleotide identity (ANI) based on a comparison of the FG106 genome to that of *P. aeruginosa* PAO1.

Metrics	FG106
	99.41
Genome A length (bp)	6,282,180
Genome B length (bp)	6,263,820
Average aligned length (bp)	4,755,752
Genome A coverage (%)	75.7
Genome B coverage (%)	75.9
Genome B:	*Pseudomonas aeruginosa* PAO1

**Table 10:** Average amino acid identity (AAI) data for the FG106 genome.

Average Amino Acid Identity (AAI)	
Metric	Sample 101
Top Hit AAI of Uniprot species	*Pseudomonas aeruginosa*
Average Amino Acid Identity (AAI)	0/998
Median amino acid identity	1
Matched fraction	0/983
Lineage	Bacteria: Proteobacteria, Gammaproteobacteria, Pseudomonadales, Pseudomonadaceae, *Pseudomonas*

## Data Availability

This project has been deposited at DDBJ/ENA/GenBank under the accession JAJNEF010000000. Raw sequence data can be found in the NCBI Sequence Read Archive, with accession number BioProject: ID PRJNA767521.

## Supplementary Materials

The following are available online at https://www.mdpi.com/article/10.3390/biology11010140/s1, Table S1. Sheet 1: *P. aeruginosa* FG106-16S rRNA sequence, Sheet 2: Comparative analysis of virulence factors and antagonistic activity related genes of *Pseudomonas aeruginosa*, FG106 with other *Pseudomonas aeruginosa* strains.
